# Gram-Negative Colonization and Bacterial Translocation Drive Neonatal Sepsis in the Indian Setting

**DOI:** 10.1007/s44197-024-00303-8

**Published:** 2024-09-30

**Authors:** Faiza Iqbal, Apurv Barche, Padmaja A. Shenoy, Leslie Edward S. Lewis, Jayashree Purkayastha, K. E. Vandana

**Affiliations:** 1 Department of Pediatrics, Kasturba Medical College, Manipal, Manipal Academy of Higher Education (MAHE), Manipal, Karnataka 576104 India; 2 Department of Pediatrics, Neonatal/ Perinatal, UMass Memorial Medical Center, Worcester, MA USA; 3 Department of Microbiology, Kasturba Medical College, Manipal, Manipal Academy of Higher Education (MAHE), Manipal, Karnataka 576104 India

**Keywords:** Neonatal sepsis, Gut microbiome, Proteobacteria, Dysbiosis, Preterm infants, Microbial colonization

## Abstract

**Background:**

The gut microbiota, comprising billions of microorganisms, plays a pivotal role in health and disease. This study aims to investigate the effect of sepsis on gut microbiome of neonates admitted to the Neonatal Intensive Care Unit.

**Methods:**

A prospective cohort study was carried out in the NICU of tertiary care hospital in Karnataka, India, from January 2021 to September 2023. Preterm neonates with birth weight < 1500 g and gestational age < 37 weeks were recruited, excluding those with congenital gastrointestinal anomalies, necrotizing enterocolitis, or blood culture-negative infections. The study population was divided into three groups: healthy neonates (Group A), neonates with drug-sensitive GNB sepsis (Group B), and neonates with pan drug-resistant GNB sepsis (Group C). Stool samples were collected aseptically, snapped in liquid nitrogen, and stored at -80⁰C for extraction of DNA and microbiome analysis.

**Results:**

The gut microbiota of healthy neonates (Group A) was dominated by Proteobacteria (24.04%), Actinobacteria (27.13%), Firmicutes (12.74%), and Bacteroidetes (3%). Predominant genera included Bifidobacterium (55.17%), Enterobacter (12.55%), Enterococcus (50.69%), Streptococcus (7.92%), and Bacteroides (3.58%).Groups B and C, the microbiota exhibited higher Proteobacteria abundance (57.16% and 66.58%, respectively) and reduced diversity of beneficial bacteria. Notably, the presence of sepsis was associated with an increase in pathogenic bacteria and a decrease in beneficial commensal bacteria.

**Conclusion:**

Neonates with sepsis exhibited significant gut microbiome dysbiosis, characterized by increased Proteobacteria and reduced beneficial bacteria diversity. These findings highlight the potential of microbiome profiling as a diagnostic tool and underscore the importance of gut microbiota modulation in managing neonatal sepsis.

## Introduction

Gut microbiota composition gives an understanding of the development of microbial flora [1]. The immature intestines of neonates, characterized by underdeveloped immunity, barrier function, and peristalsis, make them vulnerable to inflammation and sepsis [2]. Sepsis, a highly heterogeneous and potentially fatal condition, is caused primarily by a dysregulated immune response to bacterial, viral, or fungal infections, leading to significant morbidity and mortality across all ages. In 2019, the Centre for Disease Dynamics, Economics and Policy (CDDEP) reported nearly one million neonatal deaths annually within the first four weeks of life, of which 190,000 deaths are caused by sepsis in India [3]. Broad-spectrum antibiotics used for treatment of suspected sepsis can considerbly disrupt gut microbiome development and cause antimicrobial resistance. There is bidirectional relationship between gut dysbiosis and sepsis, where dysbiosis can both result from and contribute to sepsis, signifying the need to explore these interactions for improved clinical management [4, 5]. Neonates get their initial microbiota from their mothers and environment, with significant variation by place of birth. Breastfeeding provides beneficial bacteria and oligosaccharides that support gut health, helping to prevent conditions like sepsis and necrotizing enterocolitis and promoting immune function and inflammation regulation in the first 1–2 years [6].Recent research suggests that changes in gut flora is driven by factors such as disrupted tight junction proteins, altered local immunity, and decreased protective secretions—play a critical role in increasing susceptibility to conditions like necrotizing enterocolitis (NEC) and sepsis [7]. Prolonged antibiotic use has been shown to exacerbate gut dysbiosis, further increasing the risk of these conditions [8]. Globally, antibiotic-resistant bacteria are responsible for an estimated 214,000 neonatal sepsis deaths each year [9].

**Fig. 1:**
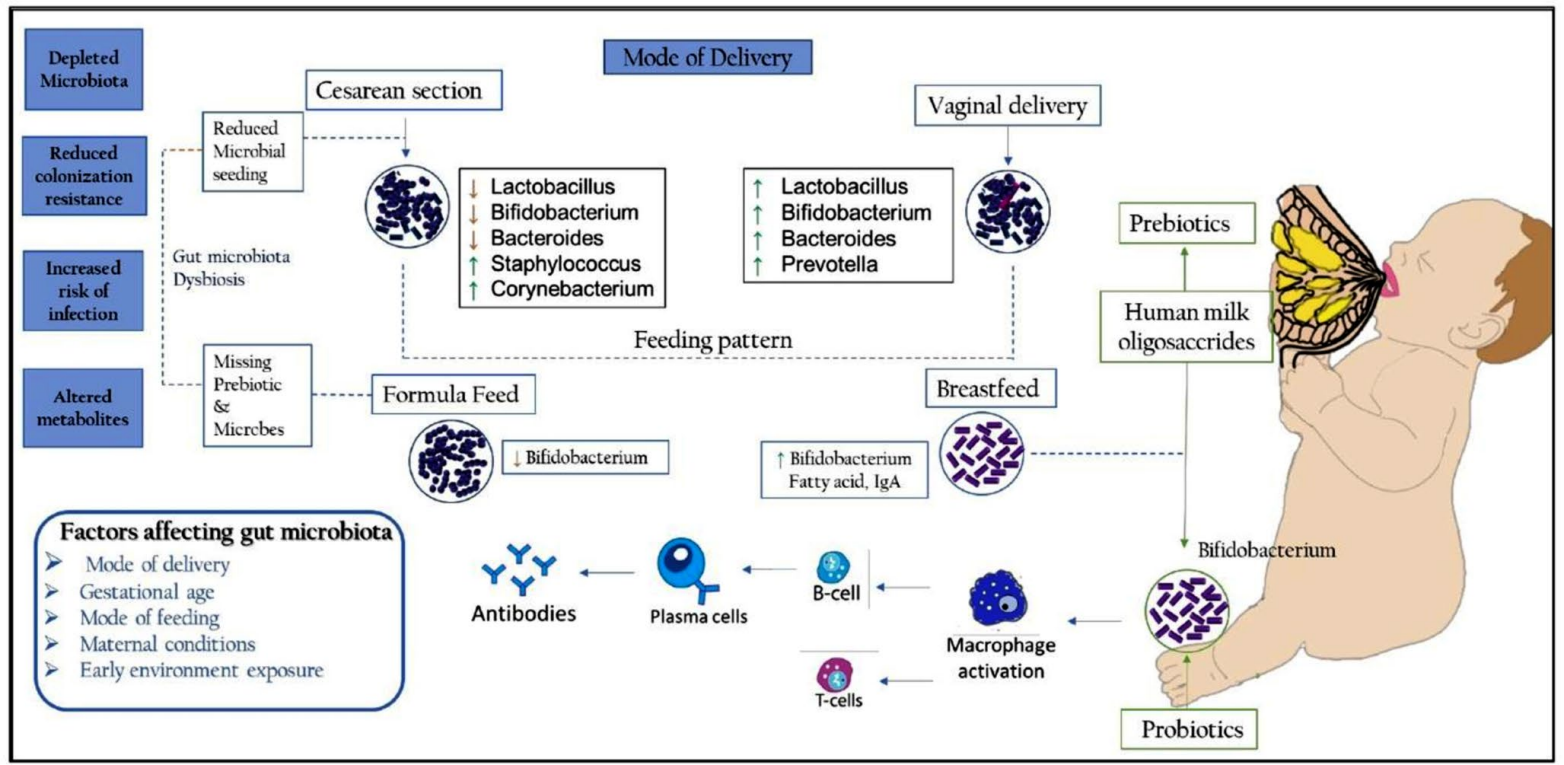
Concept of modulating gut microbiota

Gram-negative bacteria cause 18–80% of all NS [10–12]. The frequency of Gram-negative NS has increased worldwide, with an alarming increase in pan-resistant (PDR) infections [13, 14]. ‘European Centre for Disease Prevention and Control (ECDC) and the Centres for Disease Control and Prevention (CDC)’ defines PDR as ‘bacteria that have resistant to all antimicrobial agents in all antimicrobial category’ [15]. PDR infections in neonatal intestine care units are emerging as a severe problem [15]. Bacteria colonise a neonate’s gut immediately after birth. There are some potential pathogens among the hundreds of bacteria that colonize the gut. Studies have shown that Gram-negative bacilli (GNB) in the gut has been shown to predispose septicaemia in neonates [2, 16, 17]. The gut microbiota commensal may translocate into intestinal epithelium, entering the blood circulation. Some commensal gut microbes have the potential to become pathogenic. Bacteria’s like Enterobacteriaceae, Staphylococci, Enterococci, and Lactobacilli are some of the translocating bacteria. (Fig. 1).

Several factors can influence the growth and development of gut microbiota of neonate, one of which is early antibiotic treatment. Antibiotics can destroy a healthy gut microbiome bacteria in the weaning stage [18]. Early antibiotic consumption in neonates has two consequences: first, neonates may lose the advantages provided by good bacteria resulting in a decrease in commensal bacteria with delay in Bifidobacterium and Bacteroidetes colonisation [19, 20] and second, the microbial ecosystem’s balance may be shifted toward pathogenic species as a result of this disruption [21]. Antibiotics also decrease the diversity of the gut microbiota by removing sensitive strains that make up healthy flora. This can result in an overgrowth of potentially pathogenic and resistant bacteria, elevating the risk of infection, primarily with multidrug-resistant organisms [22, 23]. The extent of dysbiosis to the microbiota depends on the type and duration of the antibiotic [24]. Moreover, the gut of the neonates with sepsis loses good bacterial flora that represent a vital part of healthy neonates microbiota.

In South Asia, research from the ANISA study, reveal substantial insights into the aetiology of bacterial infections in neonates. The study revealed that despite the low attribution of bacterial infection in the group of over 63,000 live births—pathogens were detected in 46% of cases, with bacterial infections contributing to 92% of these identified causes. This underscores the impact of bacterial infections on neonatal health in South Asia, emphasing the need for focused interventions and preventive measures [25, 26]. We hypothesised a correlation between the gut microbiota and sepsis by comparing the gut microbiota in a cohort of neonates with sepsis and healthy neonates. This study aimed to investigate the gut colonisation patterns in neonates with sepsis and its correlation to determine the differences of gut microbiota between the three groups to analysed whether the gut microbiota could serve as a potential prognostic marker for neonates admitted to the neonatal intensive care unit with sepsis (NICU) using sequencing. Study also aimed to analyse if there is association between the gut microbiota and the bloodstream pathogens.

## Methodology

### Study Design and Population

**Fig. 2:**
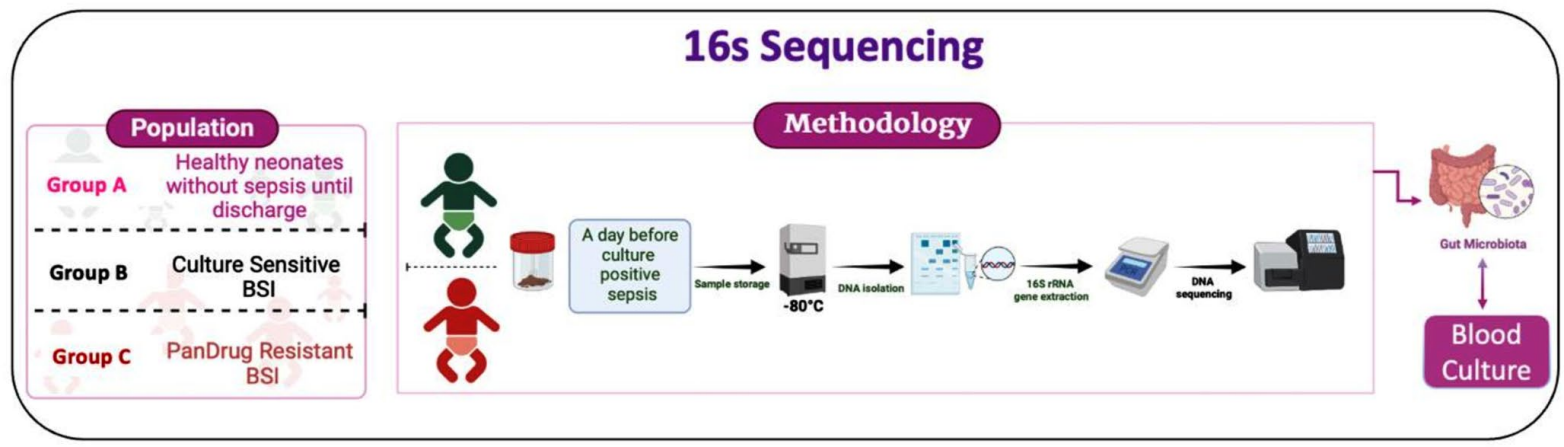
Outline of the methodology

A prospective cohort study was conducted to assess gut dysbiosis in neonates with sepsis. The study was approved by institutional ethics committee (IEC: 345/2021) and registered with CTRI (CTRI/2021/06/034251). Neonates were recruited from the NICU of a tertiary care hospital in Karnataka, India. Inborn preterm neonates with gestational age < 37 weeks and birth weights < 1500 g were enrolled after obtaining written parental consent from either parent between January 2021 and September 2023. Preterm neonates with congenital gastrointestinal anomalies, necrotizing enterocolitis, or blood culture-negative infections were excluded from study. The study participants were divided into three groups. Group A included healthy, breastfeeding neonates without sepsis until discharge. Group B comprised neonates with blood cultures showing drug-sensitive Gram-negative bacterial (GNB) infections admitted to the NICU. Group C consisted of neonates with pan drug-resistant GNB infections admitted to the NICU (Fig. 2). Demographic details of the recruited neonates, such as gestational age, gender, birth weight, and morbidities, were collected from hospital case records. Information regarding blood culture and antimicrobial susceptibility patterns was retrieved from laboratory records maintained by the hospital. Stool samples were collected aseptically using a sterile spoon from the diaper of the neonates and transferred into sterile containers.

### Sample Collection and Processing

All the eligible inborn neonates admitted to NICU were recruited at birth (day 1) and were followed prospectively, by observing them over time to identify the occurrence of sepsis. The neonates were followed prospectively for 28 days for onset of sepsis. After the follow-up period, the neonates were divided into groups based on whether they developed sepsis. Sepsis was defined as all neonates who developed Early-Onset Sepsis (EOS) or Late-Onset Sepsis (LOS) during the study period. Demographic details of the recruited neonates, such as gender, gestational age, birth weight, and morbidities, were collected from case records. Information regarding blood culture and antimicrobial susceptibility patterns was retrieved from laboratory records maintained by the hospital. For Groups B and C, stool samples were collected on the day when the gram stain of blood culture bottles showed growth of pathogenic Gram-negative bacteria, confirming sepsis. For Group A, stool samples were collected at day 14 or day of discharge whichever is earlier. Stool samples were collected aseptically using a sterile spoon from the diaper of the neonates and transferred into sterile containers. The stool samples were promptly snap-frozen in liquid nitrogen and stored at -80⁰C until DNA extraction.

### DNA Extraction and 16 S rRNA Sequencing

Genomic DNA extraction was performed using the QIAamp PowerFecal DNA Kit (Qiagen, USA) following the manufacturer’s protocol. Around 250 µg of each sample was combined with 750 µl of PowerBead Solution and 60 µl of Solution C1, then heated at 65 °C for 10 min. The mixture was Consequently vortexed with a PowerLyser Homogenizer at 1,000 RPM for 10 min. The DNA was then extracted, washed, and eluted with nuclease-free water as per the kit instructions. The extracted DNA was kept at -20 °C. The quality and concentration of purified DNA were assessed using a Qubit 4 Fluorometer with a high-sensitivity dsDNA assay (Thermo Fisher Scientific). For 16 S rRNA amplicon sequencing, the Illumina MiSeq platform (Illumina, Inc., USA) was utilized, targeting the V3-V4 region of the bacterial 16 S rRNA gene. The raw sequencing reads (fastq files) underwent adapter sequence removal and low-quality base trimming using the Trim Galore tool. The processed reads were imported into MOTHUR, and they were aligned to form contigs. These contigs were then classified into taxonomic outlines using GREENGENES v.13.8–99 database and clustered into Operational Taxonomic Units (OTUs) with estimated abundances.

### Statistical Analysis and Bioinformatic Processing

Patient demographics and susceptibility patterns of bacterial isolates were reported using mean (± SD), median (± IQR) and percentages values. The mean ± SD compiles data for continuous variables with normal distribution, while the median and range were used for skewed distribution data. Microbiota composition was assessed by abundance, diversity, and dissimilarity. Bar plots showed the top 10 phyla, genera, and species, while heatmaps also displayed top 10 and significant OTUs. The Shannon index measured with alpha diversity analysed in R using the vegan package and visualized with ggplot2. Beta diversity analysis, performed with STAMP software, utilized cosine distances and principal coordinate analysis (PCoA) for between-sample comparisons.

## Results

### Clinical Characteristics of Study Participants

The demographic details of all three cohorts are compiled in Table 1. The mean birth weights were 1926.43 ± 732.01 g in Group A, 1627.14 ± 470.81 g in Group B, and 1763.28 ± 896.79 g in Group C. The recruitment age of the neonates in the control group was 11.28 ± 2.6 days (mean ± SD).The duration of hospitalization was longer for Groups B and C (30 [20–34] days vs. 37 [27–42] days) compared to Group A (11 [7–21] days). All 14 cases of neonatal sepsis showed positive for a single pathogen as determined by blood culture, including *Klebsiella pneumoniae* 10 (71.4%), *Acinetobacter nosocomialis 1*(7.1%), *Escherichia coli* 2 (14.3%), and *Enterobacter hormaechei* 1 (7.1%).

**Table 1:** Demographic characteristics

Patient characteristics	Healthy neonates (Group A) (*n* = 7)	Sensitive neonatal sepsis (Group B) (*n* = 7)	Resistant neonatal sepsis (Group C) (*n* = 7)
Age of Diagnosis(days)^a^ Birthweight(grams)^a^	- 1926.43 ± 732.01	5 ± 3.7 1627.14 ± 470.81	7.14 ± 4.4 1763.28 ± 896.79
Sex, *n*(%)			
Female	3(43)	5(72)	7(100)
Male	4(57)	2(28)	0(0)
**Maternal parity n(%)**			
Primigravida	4(57)	4(57)	5(71)
Multigravida	3(43)	3(43)	2(29)
**Gestational Age**,** n(%)**			
Extremely Preterm (< 28 Weeks)	0(0)	0(0)	0(0)
Very Preterm (28 to 32 Weeks)	2(28)	2(28)	1(14)
Moderate Preterm(32–33 Weeks)	1(14)	0(0)	3(43)
Late Preterm (34–37 Weeks)	2(28)	5(72)	1(14)
Term > 37 Weeks	2(28)	0(0)	2(28)
**Neonatal growth outcome**,** n (%)**			
LGA	1(14)	0(0)	1(14)
AGA	5(72)	3(43)	3(43)
SGA	1(14)	4(57)	3(43)
**Mode of delivery**,** n(%)**			
Vaginal Delivery	3(43)	2(28)	2(28)
Caesarean delivery	4(57)	5(72)	5(72)
**Neonatal sepsis**,** n (%)**			
EOS	-	6(86)	4(57)
LOS **Hospitalisation days** ^**b**^	- 11(7–21)	1(14) 30(20–34)	3(43) 37(27–42)
**Neonatal Morbidities**,** n(%)**			
Bradycardia	1(14)	1(14)	3(43)
Respiratory distress	4(57)	5(72)	7(100)
Apnoea	0(0)	3(43)	1(14)
Metabolic acidosis	1(14)	0(0)	4(57)

^a^ Presented as mean ± standard deviation, ^b^ Presented as median and interquartile range, IQR

SD = Standard Deviation; IQR = Interquartile range; LGA = Large for gestational age; AGA = Accurate for gestational age; SGA = Small for gestational age; EOS = Early-onset sepsis; LOS = late-onset sepsis

### Association Between Gut Dysbiosis and Neonatal Sepsis

#### Alpha and Beta Diversity

**Fig. 3:**
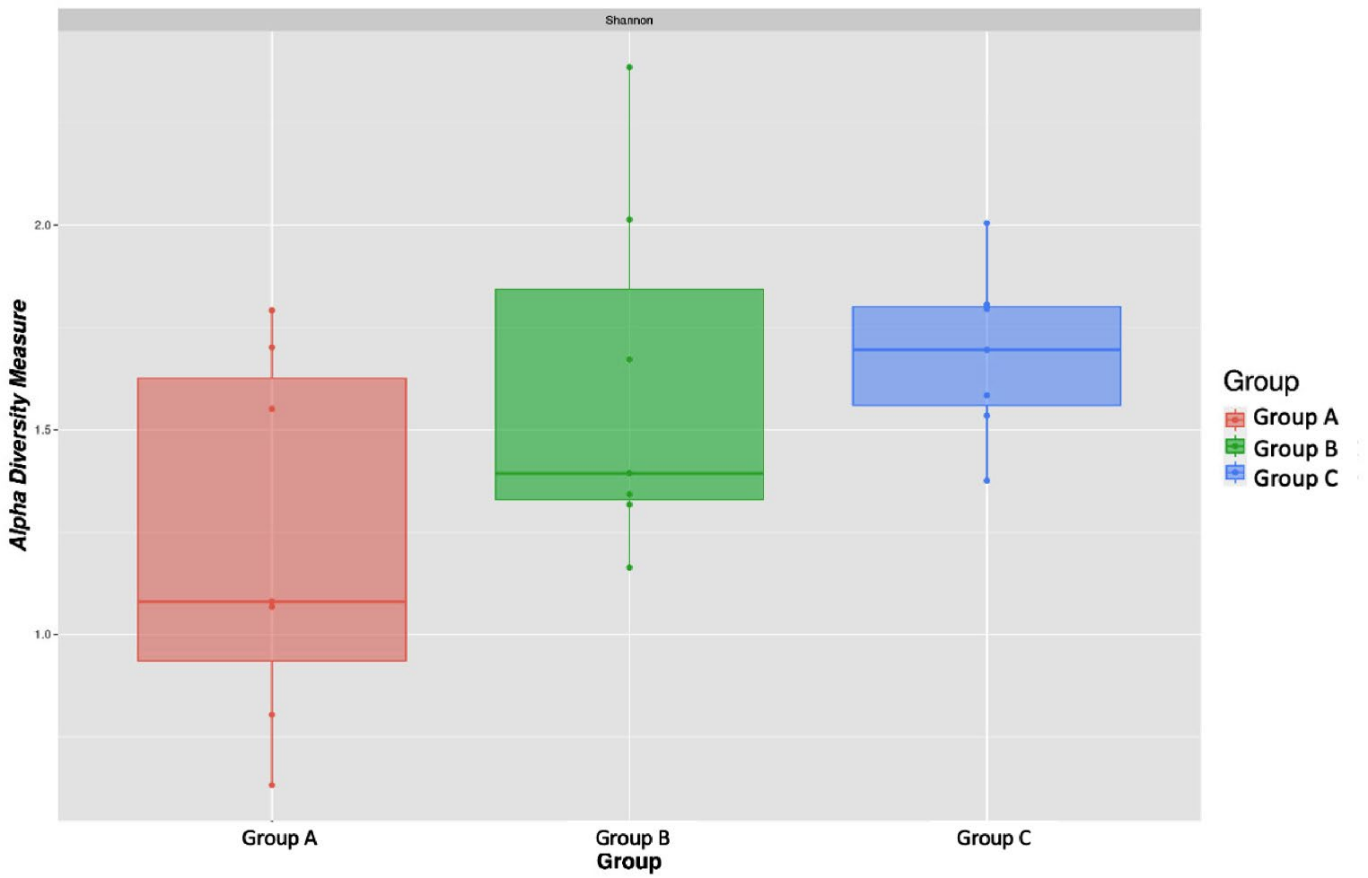
Microbial α-diversity indexes. Box plots depict differences in the fecal microbiome diversity indexes between the sepsis and control groups according to the Shannon index based on the OTU count

**Fig. 4:**
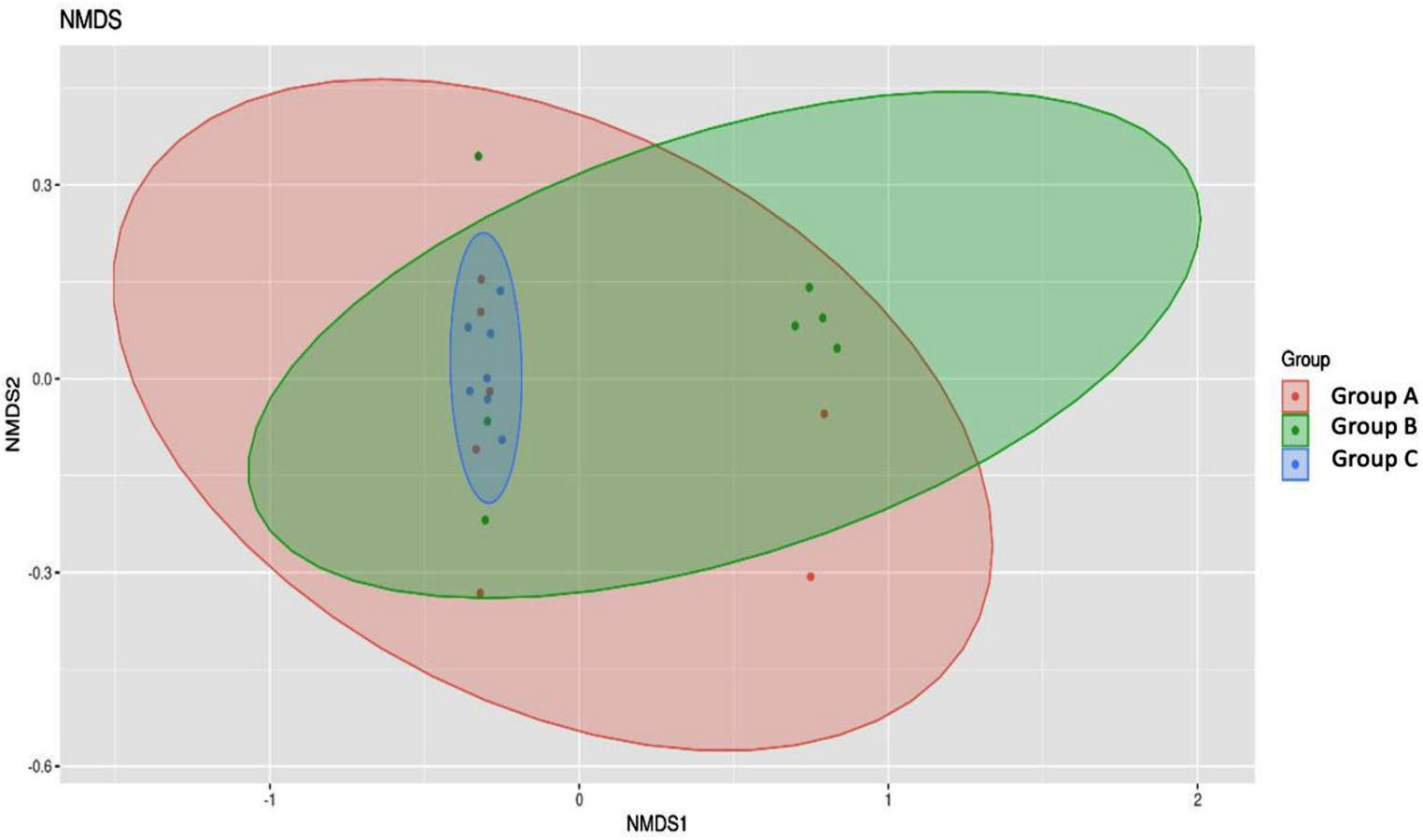
Differences in microbial composition in the three groups. A Nonmetric multidimensional scaling (NMDS) of the Whites-nonparametric-t-test indexes of samples is shown

The mean community diversity index of neonates with resistant neonatal infection (Group C) had significantly lower α-diversity, while neonates without infection (Group A) showed higher microbial diversity in the gut (Fig. 3), indicating that gut microbiota richness in healthy neonates was substantially higher than that in neonates with sepsis. The Shannon index considers both species richness and evenness. Group A shows the highest microbial diversity with a mean Shannon index of 2.166. Group B and group C exhibit lower diversity with mean values of 1.773 and 1.949, respectively. This indicates that healthy neonates have a more diverse and evenly distributed gut microbiota compared to those with sepsis. A substantial difference was also identified in β-diversity (Whites non-parametric t-test) based on the operational taxonomical unit (OTUs) of both sepsis groups (Group B and Group C) when compared to the Group A with ( *p* < 0.005) among each group, indicating a significant difference in microbial community structure between sepsis group and control group, as depicted in Fig. 4; Table 2.

**Table 2:** Comparison between different cohorts to measure β-diversity using whites-nonparametric-t-test

Comparison	Significant OTUs(*p* value < = 0.05)
Cohort1 vs. Cohort2	0.026371192
Cohort1 vs. Cohort3	0.015968461
Cohort2 vs. Cohort3	0.024628583

### Alternation in the Taxa

**Fig. 5:**
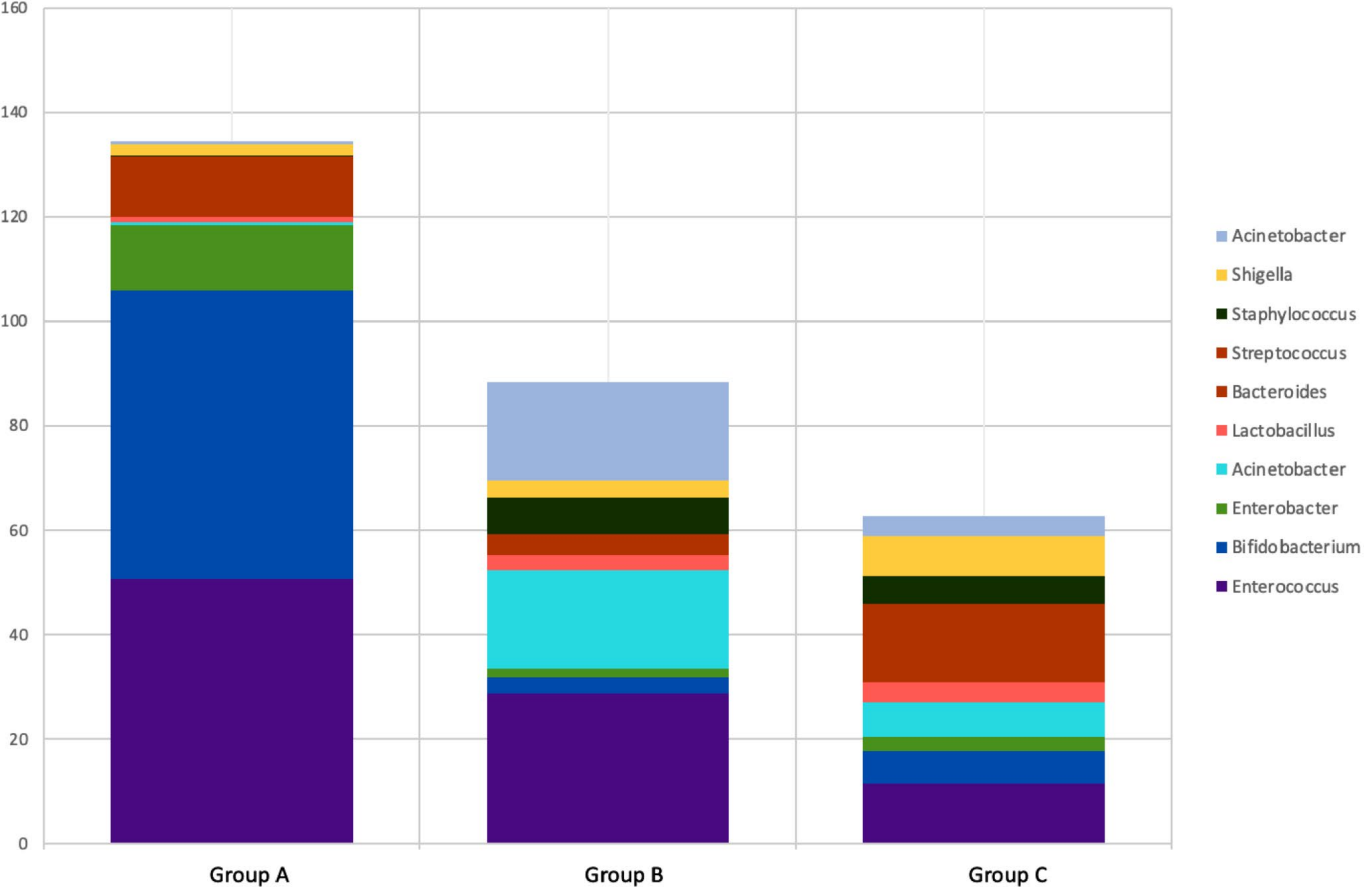
High correlation microbiota composition and pathogens identified by sequencing. Each bar represent the microbiota composition of each of the group of at genus level

The analysis revealed the presence of several OTUs associated with bacterial families known for pathogenic potential. Notably, the most prevalent species was OTU 001 (Proteobacteria; Gammaproteobacteria; Enterobacteriales), which exhibited mean relative frequencies of 42.74% and 53.10% in Group B and Group C, respectively. Additionally, OTU003 (Firmicutes; Bacilli; Lactobacillales; Enterococcaceae) was significantly present, with a frequency of 24.75% in Group B and 12.30% in Group C. These data suggest a microbial composition with potential clinical significance, as some identified families, such as Enterococcaceae, Enterobacteriales and Streptococcaceae, are known to encompass bacteria with pathogenic potential. These findings underscore the importance of monitoring gut microbiome profiles in preterm neonates for early detection and potential intervention to prevent bloodstream infections. The phylum-level composition of the gut microbiota in neonatal cohorts is represented as a heat map in Fig. 5. We meticulously tracked the relative abundance of causative species associated with sepsis. Remarkably, in neonates with Enterobacteriaceae sepsis, the causative species constituted more than 10% of the gut microbiota. Furthermore, in 75% of the neonates, the relative abundance of the sepsis-causing Enterobacteriaceae species in the stool was even more pronounced, exceeding 45%.

The major phyla in group A were Proteobacteria (24.04%), Actinobacteria(27.13%), Firmicutes(12.74%) and Bacteroidetes (3%).At genus level, top five predominant genera in group A were Bifidobacterium(55.17%), Enterobacter(12.55%), Enterococcus (50.69%), Streptococcus(7.92%) and Bacteroides(3.58%), while in the group B and group C, the predominant genera were Enterococcus (28.9% vs. 11.4%, respectively), Bifidobacterium(2.9% and 6.4%, respectively), Bacteroides(1.86% vs. 1.44%, respectively) and Acinetobacter (18.9% vs. 6.8%, respectively). The gut microbiome in group A was constituted rich and diverse bacteria such as the genera Enterobacter and Enterococcus, compared with the other groups. The genus Bifidobacterium accounted for 55.17%, 2.95%, and 6.41% in group A, group B and group C, respectively. The genus Enterococcus accounted for 50.69%, 28.9%, and 11.41% in group A, B and C, respectively. The relatively high abundance of the phylum Proteobacteria was observed in the group B and group C (57.16% vs. 66.58%, respectively), compared to the healthy groups (group A). Thus the presence of sepsis was associated with a significant reduction in beneficial and non-pathogenic bacteria such as Bifidobacterium, Enterococcus and Bacteroides in the gut of the neonates.

### Potentially Pathogenic Bacterial Strains Observed in the Gut Microbiota and Their Correlations with Bloodstream Pathogens

Neonates, particularly preterm infants, experience a established progression of gut colonization, starting with the dominance of aerobic organisms such as *E. coli and Klebsiella pneumoniae*, followed by a transformation to microaerophilic and finally strict anaerobic species. While these early colonizers are part of normal microbiota, they can also act as opportunistic pathogens in certain circumstances, especially in the vulnerable population of preterm neoantes in the NICU. A total of 71.4% [10] of the blood pathogens present in the blood were also detected in the stool. In this study, it was observed that neonates with *E. coli* and *Klebsiella pneumoniae* bloodstream infections seemed to have concomitant gut colonisation, implying that the gut could be a source of these infections. As such, while these bacteria are often present during early colonization and do not inherently indicate pathogenicity, disruptions in the typical colonization process, such as a delayed or insufficient colonization by beneficial bacteria like *Lactobacillus spp* and *Bifidobacterium spp* which may shift the balance towards pathogenicity. While the data indicate that the gut is a potential source of sepsis-causing pathogens, further investigation is needed to confirm whether the same pathogenic strains colonize multiple body sites.

## Discussion

Disruption of the gut microbiome emerges to be risk factor for sepsis and consequent organ failure. Potentially pathogenic bacteria may reside in intestinal lumen of healthy neonates without being able to proliferate, spread, and cause disease [27, 28]. Any dysbiosis in the gut may cause loss of protective flora allowing the proliferation of potentially pathogenic flora into pathogenic pathobionts. In present study, that neonates with sepsis have profoundly distorted and dysbiosis composition of protective commensals of the gut microbiota. Due to the immature intestine of a preterm neonate, which includes underdeveloped immunity, barrier function, and peristalsis, they are predisposed to infection and inflammation. One condition that may result from alterations in the gut microbiota is neonatal sepsis. This condition increases the risk of early colonization by pathogenic microbiota, leading to dysbiosis of gut, a dysregulated immune response, organ dysfunction, and potentially fatal outcomes [29]. The microbial composition is impacted by maternal health and habits and significantly impacts pregnancy and fetal outcomes [30]. In present study observed that in gut microbiota of neonates with sepsis was significantly different from that of healthy neonates without sepsis. In healthy neonates the gut microbiota composition was dominated by the *Proteobacteria*, *Bacteroidetes*, *Firmicutes*, and *Actinobacteria*. The gut flora of sepsis neonates included a low diversity of microbiota with more potentially pathogenic bacteria and fewer good commensal bacteria than in healthy neonates. The abundance of *Proteobacteria* in neonates with sepsis (Group B and C) increased significantly when compared to the healthy neonates. Proteobacteria abundance has been proposed as a diagnostic microbial signature of dysbiosis, epithelial malfunction, and disease risk [31].In this study, we also found that there was significant increase in the phylum *Proteobacteria* compared to the healthy controls. Proteobacteria are opportunistic Gram-negative pathogens that produce potent inflammatory chemicals such as lipopolysaccharide.

Numerous literatures supports that Proteobacteria blooms indicate dysbiosis or an unstable gut microbial composition [27, 31]. These notions were supported by the literature of other study: the neonates with sepsis have a lower diversity of microbiota than healthy neonates [24, 32–34]. Lee et al. (2021) in his study observed that the gut microbiome composition of preterm infants and healthy neonates was similar up to few weeks after birth, which then later evolved toward dysbiosis of the flora due to increasing *Proteobacteria* and decreasing *Firmicutes* due to the onset of sepsis [32]. Madan et al. (2013) demonstrated that the neonates who developed sepsis had an increase in the abundance of *Staphylococcus*, while in healthy neonates developed rich microbial diversity with predominance of *Klebsiella*,* Clostridium*, and *Veillonella* [35]. It is important to note that organisms such as *E. coli* and *Klebsiella spp*, while generally considered part of the normal gut flora, can become pathogenic under certain circumstances, particularly in immune naïve neonates. These bacteria are not always pathogenic unless they have specific virulence traits; however, in the context of neonatal immune immaturity and bacterial translocation, they can also lead to severe infections, as observed in our study [36].In our study, we observed a gradual reduction in beneficial bacteria such as *Lactobacillus spp* and *Bifidobacterium spp* in neonates with sepsis, with *Bifidobacterium spp* accounting for only 2.9% in Group B and 6.4% in Group C, compared to 55.17% in healthy neonates (Group A). Similarly, *Enterococcus spp* levels were significantly lower in the sepsis groups (28.9% in Group B, 11.41% in Group C) compared to 50.69% in the healthy group. This dysbiosis of microbiota composition, particularly delayed colonization by beneficial bacteria, may increase the risk of sepsis, as also observed by, who reported a similar reduction of *Bifidobacterium spp* in preterm infants with late-onset sepsis, where levels dropped to 3–6% in infected infants compared to over 40% in healthy controls [37].

In this study, we observed significant differences in the abundance and timing of key beneficial bacterial genera, particularly *Bifidobacterium spp*, which is known to play a important role in maintaining gut health and supporting immune function in neonates. In Group A (average age of stool sample collection: 11.28 ± 2.6 days), *Bifidobacterium spp* was the predominant genus, accounting for 55.17% of the microbiota. In contrast, Group B (average age 5 ± 3.7 days) and Group C (average age 7.14 ± 4.4 days) showed lower relative abundances of *Bifidobacterium spp*, at 2.95% and 6.41%, respectively. The earlier and more abundant colonization by *Bifidobacterium spp* in Group A suggests a protective role against dysbiosis and sepsis, supporting existing literature on the beneficial effects of early colonization by this genus. Sequencing of the stool samples demonstrated that gut microbiota richness and evenness measured by α-diversity (Shannon index) in neonates with sepsis were significantly lower in group C (*R* = 2.16) and Group B group (*R* = 1.9), compared the group A (1.7). The overall microbial composition indicated by the β-diversity index was significantly and substantially diverse with more healthier gut flora in HS group than in neonatal sepsis group (group B and C). These observations were consistent with previous research by Dul et al. 2021, who reported diverse and rich gut microbiomes in healthy neonates (*P* < 0.001) and significantly low diversity in sepsis neonates [38]. Madan et al. 2013 also had similar findings [35]. Lee et al. (2021) in his study concluded that preterm neonates with gut dysbiosis are at risk of sepsis due to horizontal transmission of potential pathogens from the gut [32]. Our study adds to this growing body of evidence by showing that 71.4% of bloodstream pathogens found in septic neonates were also detected in the gut, suggesting that early dysbiosis may play a role in the translocation of bacteria leading to sepsis. These findings emphasize the importance of promoting early colonization with beneficial microbes as a potential intervention to reduce the risk of neonatal infections.

The gut microbiome profiles identified in this study have potential use as biomarkers for early diagnosis and prognosis of neonatal sepsis. Significant differences in microbiome composition between healthy and septic neonates could aid in developing predictive models for sepsis risk, allowing for timely and targeted interventions. Additionally, Therapeutic strategies aimed at restoring a healthy microbiome, such as probiotic supplementation or microbiome transplantation, hold promise for improving outcomes in septic neonates. Recent studies have supported the use of probiotics to modify gut microbiota and reduce the risk of neonatal sepsis. A systematic review by et al. Deshmukh & Patole, 2021 of 30 non-randomized studies involving over 77,000 neonates showed a 15% reduction in late-onset sepsis (LOS) with routine probiotic supplementation [39]. Moreover, a study on extremely preterm infants showed that probiotic supplementation reduced the rate of LOS from 70% in the control group to 47.1% in the probiotic group (*p* = 0.015), along with increasing Lactobacillus abundance [40]. As research unfolds, integrating microbiome-based strategies into neonatal care may significantly enhance sepsis management and improve clinical outcomes for vulnerable infants.

### Global Burden of Neonatal Sepsis: Understanding Changes in the Microbiota as a Link to Prevention

Neonatal sepsis persists to be a severe health challenge in India, aiding significantly to increase neonatal mortality and morbidity. In low- and middle-income regions, particularly in the Indian setting, up to 60% of sepsis cases are attributed to Gram-negative bacteria such as *K. pneumoniae* and *Escherichia coli* [41]. The widespread prevalence of these pathogens is exacerbated by growing resistance to most common antibiotics, including aminoglycosides and third-generation cephalosporins, with resistance rates ranging from 42–69% [35]. Despite the crucial nature of this issue, efforts to prevent or reduce neonatal sepsis remain limited. The excessive dependence on broad-spectrum antibiotics, often used indiscriminately, contributes to the increasing problem of antimicrobial resistance, which further complicates treatment options [42].Additionally, prolonged antibiotic use disrupts the neonatal gut microbiota, a key factor in sepsis pathogenesis. This disruption promotes bacterial translocation, allowing Gram-negative bacteria to spread from the gut to the bloodstream, profoundly contributing to sepsis [43]. Understanding the dynamics of neonatal microbiota and its role in sepsis offers new avenues for intervention. By focusing on maintaining or restoring a healthy microbiota, through interventions like probiotics or microbiota modulation, it may be possible to decrease translocation of bacteria and sepsis risk [35]. This approach holds the potential to save millions of lives and significantly reduce economic burdens in the Indian healthcare system.

### Advantages and Limitations of the Study

This study provides valuable insights into the gut microbiome composition of neonates with sepsis, emphasizing significant alterations when compared to healthy neonates. The detailed microbial profiling aid in the identification of specific bacterial genera involved with health and disease states, potentially helping in the development of predictive models and targeted therapies. Additionally, the study’s focus on drug-resistant pathogens highlights the relevance of findings in the context of rising antimicrobial resistance. However, there are notable limitations. The relatively small cohort size may limit the generalizability of the findings, requiring the need for larger studies for validation. The present study also did not assess the functional implications of the altered microbiome, which could provide deeper insights into the mechanisms of sepsis-related dysbiosis.

### Future Implications

The study’s findings underscores the potential of microbiome profiling as a diagnostic tool for early identification of sepsis risk in neonates, enabling timely and targeted interventions. Therapeutic strategies such as probiotic supplementation or microbiome transplantation could help restore a healthy microbiome, reducing sepsis severity and duration. Insights from this research could also inform guidelines for antibiotic use in neonates, aiming to minimize dysbiosis and preserve beneficial bacterial populations. Future studies should focus on long term monitoring of microbiome changes and functional analyses to deepen understanding of microbiome-host interactions in neonatal sepsis.

## Conclusion

Overall, the current investigation presents significant changes in the gut microbiome of neonates with sepsis compared to healthy neonates. The presence of sepsis, particularly due to drug-resistant pathogens, is associated with marked reduction in beneficial bacteria such as *Bifidobacterium*, *Enterococcus*, and *Bacteroides*. The higher prevalence of Proteobacteria and genera like *Acinetobacter* in septic neonates highlights the impact of pathogenic infections on gut microbial composition. Our findings emphasises the importance of monitoring and potentially modulating the gut microbiome in neonates at risk of sepsis to improve clinical outcomes. Future research should explore targeted interventions to restore healthy gut microbiota and their potential therapeutic benefits in neonatal sepsis.

## Data Availability

No datasets were generated or analysed during the current study.

## Abbreviations

NICU: Neonatal Intensive Care Unit
BSI: Bloodstream Infection
GARDP: Global Antibiotic Research & Development Partnership
CDDEP: Centre for Disease Dynamics, Economics and Policy
NS: Neonatal Sepsis
GNB: Gram-Negative Bacteria
PDR: Pan Drug-Resistant
ECDC: European Centre for Disease Prevention and Control
CDC: Centers for Disease Control and Prevention
OTU: Operational Taxonomic Unit
DNA: Deoxyribonucleic Acid
rRNA: Ribosomal RNA
RNA: Ribonucleic Acid
SD: Standard Deviation
IQR: Interquartile Range
LGA: Large for Gestational Age
AGA: Accurate for Gestational Age
SGA: Small for Gestational Age
EOS: Early-Onset Sepsis
LOS: Late-Onset Sepsis
NMDS: Nonmetric Multidimensional Scaling
PCoA: Principal Coordinate Analysis
QIAamp: QIAGEN DNA Extraction Kit Brand
MALDI-TOF: Matrix-Assisted Laser Desorption/Ionization-Time of Flight
CLSI: Clinical and Laboratory Standards Institute
